# 6-Methyl­nicotinic acid

**DOI:** 10.1107/S1600536811031837

**Published:** 2011-08-17

**Authors:** Mei-Ling Pan, Yang-Hui Luo, Shu-Lin Mao

**Affiliations:** a Ordered Matter Science Research Center, College of Chemistry and Chemical Engineering, Southeast University, Nanjing 210096, People’s Republic of China

## Abstract

All non-H atoms of the title compound, C_7_H_7_NO_2_, are nearly coplaner, the r.m.s. deviation being 0.0087 Å. In the crystal, the partially overlapped arrangement and the face-to-face distance of 3.466 (17) Å between parallel pyridine rings of neighboring mol­ecules indicates the existence of π–π stacking. Inter­molecular O—H⋯N hydrogen bonding and weak C—H⋯O hydrogen bonding are present in the crystal structure.

## Related literature

The title compound is an inter­mediate of the drug etoricoxib (systematic name: 5-chloro-6′-methyl-3-[4-(methyl­sulfon­yl)phen­yl]- 2,3′-bipyridine). For the structure of etoricoxibium picrate, see: Jasinski *et al.* (2011 ▶).
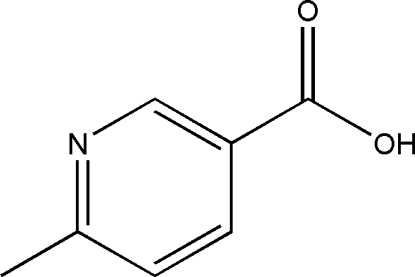


## Experimental

### 

#### Crystal data

C_7_H_7_NO_2_
*M* *_r_* = 137.14
Monoclinic, 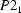

*a* = 3.8788 (8) Å
*b* = 13.634 (3) Å
*c* = 6.1094 (12) Å
β = 90.51 (3)°
*V* = 323.07 (12) Å^3^
*Z* = 2
Mo *K*α radiation
μ = 0.11 mm^−1^
*T* = 293 K
0.20 × 0.20 × 0.20 mm

#### Data collection

Rigaku SCXmini diffractometer
3358 measured reflections
763 independent reflections
634 reflections with *I* > 2σ(*I*)
*R*_int_ = 0.059

#### Refinement

*R*[*F* ^2^ > 2σ(*F* ^2^)] = 0.049
*wR*(*F* ^2^) = 0.126
*S* = 1.05
763 reflections
92 parameters
1 restraint
H-atom parameters constrained
Δρ_max_ = 0.25 e Å^−3^
Δρ_min_ = −0.16 e Å^−3^

### 

Data collection: *CrystalClear* (Rigaku, 2005 ▶); cell refinement: *CrystalClear*; data reduction: *CrystalClear*; program(s) used to solve structure: *SHELXS97* (Sheldrick, 2008 ▶); program(s) used to refine structure: *SHELXL97* (Sheldrick, 2008 ▶); molecular graphics: *DIAMOND* (Brandenburg & Putz, 2005 ▶); software used to prepare material for publication: *SHELXL97*.

## Supplementary Material

Crystal structure: contains datablock(s) I, global. DOI: 10.1107/S1600536811031837/xu5270sup1.cif

Structure factors: contains datablock(s) I. DOI: 10.1107/S1600536811031837/xu5270Isup2.hkl

Supplementary material file. DOI: 10.1107/S1600536811031837/xu5270Isup3.cml

Additional supplementary materials:  crystallographic information; 3D view; checkCIF report

## Figures and Tables

**Table 1:** Hydrogen-bond geometry (Å, °)

*D*—H⋯*A*	*D*—H	H⋯*A*	*D*⋯*A*	*D*—H⋯*A*
O1—H1⋯N1^i^	0.82	1.87	2.664 (4)	163
C4—H4*A*⋯O2^ii^	0.93	2.54	3.350 (4)	146

Symmetry codes: (i) 
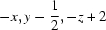
; (ii) 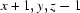.

**Fig. 1.**
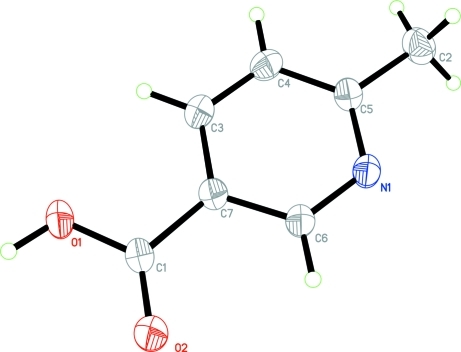
The molecular structure of the title compound with atom-numbering scheme. Displacement ellipsoids are drawn at the 30% probability level. H atoms are represented as small spheres of arbitrary radii.

**Fig. 2.**
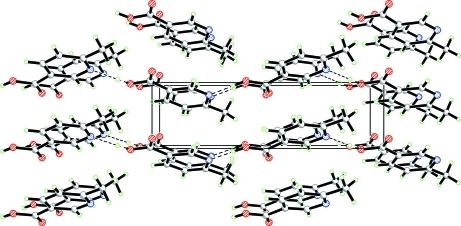
Packing diagram.

## supplementary crystallographic information

### Comment

The title compound is the drug intermediate of etoricoxib (a non- steroidal anti-inflammatory drug for the treatment of arthritis and osteoarthritis) (Jasinski *et al.*, 2011). As part of our interest in the intermediate, we report here the crystal structure of the title compound.

The molecular structure of the title compound is shown in Fig. 1. All the non-H atoms are almost located in one plane as the atoms O1 and O2 are shifted 0.0377 and 0.0236 Å out of the pyridine ring plane, respectively.

The crystal structure is stabilized by intermolecular O—H···N and C—H···O hydrogen bonds (Table 1). π···π stacking is present between pyridine rings of the neighboring molecules.

### Experimental

6-Methyl-nicotinic acid was purchased commercially. Crystals suitable for X-ray diffraction were obtained by slow evaporation of a methanol solution.

### Refinement

All H atoms were fixed geometrically and treated as riding with C—H (CH_3_) = 0.96 Å or C—H (CH) = 0.93 Å and O—H = 0.82 Å with *U*_iso_(H) =1.5 *U*_eq_(C,O) for methyl and carboxyl H atoms and *U*_iso_(H) = 1.2*U*_eq_(C) for the others. Friedel pairs were merged as no significant anomalous scatterings.

### Crystal data

**Table:**

C_7_H_7_NO_2_	*F*(000) = 144
*M**_r_* = 137.14	*D*_x_ = 1.410 Mg m^−^^3^
Monoclinic, *P*2_1_	Mo *K*α radiation, λ = 0.71073 Å
Hall symbol: P 2yb	Cell parameters from 764 reflections
*a* = 3.8788 (8) Å	θ = 3.3–27.5°
*b* = 13.634 (3) Å	µ = 0.11 mm^−^^1^
*c* = 6.1094 (12) Å	*T* = 293 K
β = 90.51 (3)°	Prism, colorless
*V* = 323.07 (12) Å^3^	0.20 × 0.20 × 0.20 mm
*Z* = 2	

### Data collection

**Table:**

Rigaku SCXmini diffractometer	634 reflections with *I* > 2σ(*I*)
Radiation source: fine-focus sealed tube	*R*_int_ = 0.059
graphite	θ_max_ = 27.5°, θ_min_ = 3.3°
Detector resolution: 13.6612 pixels mm^-1^	*h* = −5→4
CCD_Profile_fitting scans	*k* = −17→17
3358 measured reflections	*l* = −7→7
763 independent reflections	

### Refinement

**Table:**

Refinement on *F*^2^	Primary atom site location: structure-invariant direct methods
Least-squares matrix: full	Secondary atom site location: difference Fourier map
*R*[*F*^2^ > 2σ(*F*^2^)] = 0.049	Hydrogen site location: inferred from neighbouring sites
*wR*(*F*^2^) = 0.126	H-atom parameters constrained
*S* = 1.05	*w* = 1/[σ^2^(*F*_o_^2^) + (0.0712*P*)^2^ + 0.0005*P*] where *P* = (*F*_o_^2^ + 2*F*_c_^2^)/3
763 reflections	(Δ/σ)_max_ < 0.001
92 parameters	Δρ_max_ = 0.25 e Å^−^^3^
1 restraint	Δρ_min_ = −0.16 e Å^−^^3^

### Special details

**Table:**

Geometry. All esds (except the esd in the dihedral angle between two l.s. planes) are estimated using the full covariance matrix. The cell esds are taken into account individually in the estimation of esds in distances, angles and torsion angles; correlations between esds in cell parameters are only used when they are defined by crystal symmetry. An approximate (isotropic) treatment of cell esds is used for estimating esds involving l.s. planes.
Refinement. Refinement of *F*^2^ against ALL reflections. The weighted *R*-factor *wR* and goodness of fit *S* are based on *F*^2^, conventional *R*-factors *R* are based on *F*, with *F* set to zero for negative *F*^2^. The threshold expression of *F*^2^ > σ(*F*^2^) is used only for calculating *R*-factors(gt) *etc*. and is not relevant to the choice of reflections for refinement. *R*-factors based on *F*^2^ are statistically about twice as large as those based on *F*, and *R*- factors based on ALL data will be even larger.

### Fractional atomic coordinates and isotropic or equivalent isotropic displacement parameters (Å^2^)

**Table:**

	*x*	*y*	*z*	*U*_iso_*/*U*_eq_	
O1	0.0195 (8)	0.40438 (18)	0.9514 (4)	0.0631 (8)	
H1	−0.0573	0.3629	1.0349	0.095*	
O2	−0.1535 (7)	0.50696 (18)	1.2089 (4)	0.0675 (8)	
C1	−0.0216 (7)	0.4918 (2)	1.0367 (5)	0.0440 (7)	
C2	0.4505 (10)	0.8121 (3)	0.5202 (7)	0.0574 (9)	
H2A	0.5640	0.8578	0.6162	0.086*	
H2B	0.2546	0.8431	0.4526	0.086*	
H2C	0.6080	0.7911	0.4092	0.086*	
C3	0.2600 (8)	0.5539 (3)	0.6920 (6)	0.0465 (8)	
H3A	0.2823	0.4902	0.6394	0.056*	
C4	0.3724 (8)	0.6311 (3)	0.5713 (5)	0.0482 (8)	
H4A	0.4753	0.6202	0.4366	0.058*	
C5	0.3345 (9)	0.7257 (3)	0.6479 (5)	0.0423 (7)	
C6	0.0802 (8)	0.6670 (2)	0.9593 (5)	0.0439 (8)	
H6A	−0.0207	0.6796	1.0940	0.053*	
N1	0.1857 (8)	0.74310 (17)	0.8408 (4)	0.0453 (7)	
C7	0.1119 (9)	0.5709 (2)	0.8942 (5)	0.0395 (7)	

### Atomic displacement parameters (Å^2^)

**Table:**

	*U*^11^	*U*^22^	*U*^33^	*U*^12^	*U*^13^	*U*^23^
O1	0.1000 (19)	0.0295 (13)	0.0602 (16)	−0.0059 (12)	0.0191 (14)	0.0014 (11)
O2	0.107 (2)	0.0408 (16)	0.0551 (13)	−0.0023 (14)	0.0297 (14)	−0.0016 (11)
C1	0.0556 (18)	0.0312 (16)	0.0452 (16)	−0.0007 (15)	0.0018 (14)	0.0039 (14)
C2	0.063 (2)	0.048 (2)	0.0615 (19)	−0.0032 (17)	0.0127 (17)	0.0114 (17)
C3	0.054 (2)	0.0369 (18)	0.0483 (15)	0.0028 (14)	0.0021 (14)	−0.0069 (14)
C4	0.0549 (17)	0.047 (2)	0.0432 (16)	0.0036 (15)	0.0089 (13)	−0.0069 (15)
C5	0.0446 (16)	0.0364 (17)	0.0460 (16)	0.0002 (14)	0.0013 (13)	0.0022 (14)
C6	0.0542 (19)	0.0347 (18)	0.0431 (16)	0.0006 (14)	0.0076 (15)	0.0002 (13)
N1	0.0578 (16)	0.0310 (16)	0.0472 (14)	0.0019 (12)	0.0085 (13)	−0.0009 (10)
C7	0.0437 (16)	0.0338 (17)	0.0409 (15)	0.0005 (11)	−0.0005 (13)	0.0026 (11)

### Geometric parameters (Å, °)

**Table:**

O1—C1	1.311 (4)	C3—C7	1.387 (4)
O1—H1	0.8200	C3—H3A	0.9300
O2—C1	1.192 (3)	C4—C5	1.380 (5)
C1—C7	1.482 (4)	C4—H4A	0.9300
C2—C5	1.485 (5)	C5—N1	1.337 (4)
C2—H2A	0.9600	C6—N1	1.332 (4)
C2—H2B	0.9600	C6—C7	1.375 (5)
C2—H2C	0.9600	C6—H6A	0.9300
C3—C4	1.359 (5)		
			
C1—O1—H1	109.5	C3—C4—C5	120.3 (3)
O2—C1—O1	124.3 (3)	C3—C4—H4A	119.9
O2—C1—C7	123.2 (3)	C5—C4—H4A	119.9
O1—C1—C7	112.6 (2)	N1—C5—C4	120.8 (3)
C5—C2—H2A	109.5	N1—C5—C2	117.2 (3)
C5—C2—H2B	109.5	C4—C5—C2	121.9 (3)
H2A—C2—H2B	109.5	N1—C6—C7	123.8 (3)
C5—C2—H2C	109.5	N1—C6—H6A	118.1
H2A—C2—H2C	109.5	C7—C6—H6A	118.1
H2B—C2—H2C	109.5	C6—N1—C5	118.6 (3)
C4—C3—C7	119.4 (3)	C6—C7—C3	117.1 (3)
C4—C3—H3A	120.3	C6—C7—C1	119.4 (3)
C7—C3—H3A	120.3	C3—C7—C1	123.5 (3)
			
C7—C3—C4—C5	−1.0 (4)	N1—C6—C7—C1	−178.6 (3)
C3—C4—C5—N1	−0.3 (5)	C4—C3—C7—C6	1.4 (4)
C3—C4—C5—C2	−179.4 (4)	C4—C3—C7—C1	179.3 (3)
C7—C6—N1—C5	−0.7 (5)	O2—C1—C7—C6	−1.0 (5)
C4—C5—N1—C6	1.2 (5)	O1—C1—C7—C6	178.6 (3)
C2—C5—N1—C6	−179.7 (4)	O2—C1—C7—C3	−178.8 (3)
N1—C6—C7—C3	−0.6 (5)	O1—C1—C7—C3	0.7 (4)

### Hydrogen-bond geometry (Å, °)

**Table:**

*D*—H···*A*	*D*—H	H···*A*	*D*···*A*	*D*—H···*A*
O1—H1···N1^i^	0.82	1.87	2.664 (4)	163
C4—H4A···O2^ii^	0.93	2.54	3.350 (4)	146

Symmetry codes: (i) −*x*, *y*−1/2, −*z*+2; (ii) *x*+1, *y*, *z*−1.
